# Introductory Discourse on the Mode of Investigating the Sciences Belonging to the Medical Profession, Delivered in the Theatre of St. George's Hospital, October 1, 1846

**Published:** 1847-01

**Authors:** 


Art. VI.—1. *Introductory Discourse on the mode of Investigating the Sciences belonging to the Medical Profession, delivered in the Theatre of St. George's Hospital, October* 1, 1846. By Sir Benjamin C. Brodie, Bart., &c.—*London,* 1846. 8vo, pp. 13.

2. *On Medical Education: being a Lecture delivered at King's College, London, at the opening of the Medical Session,* 1846-7, *to which is added a Lecture delivered on the same occasion in the year,* 1842. By William A. Guy, m.b., Cantab., Professor of Forensic Medicine, King's College, &c.—*London,* 1846. 8vo, pp. 64.

3. *The Motives of Industry in the Study of Medicine: an address delivered at St. Bartholomew's Hospital, on Thursday, October* 1, 1846. By James Paget, f.r.c.s., Warden of the College and Lecturer on Physiology in the Hospital.—*London,* 1846. 8vo, pp. 30.

1. That the readers and auditors of a long succession of introductory lectures should turn away with a sort of frigid aversion from either the sight or hearing of another, is not, perhaps, very unnatural, when we consider the elements of which they are usually composed and the small amount of variety which is generally exhibited in their construction. But there is that about a superior mind which displays itself in the mode of discharging even the most formal duties or expounding even the most common-place truths. In the three "introductories" before us, we recognize as many exemplifications of this fact, for each is distinguished by some excellence that renders it worthy of special notice. The Discourse of Sir B. Brodie is expressly addressed to the pupils of the school of which he has been so long an ornament; and it exhibits, like all his addresses on medical and surgical topics, his strong practical sense, and his earnest desire that his hearers should be trained in habits of correct observation and logical deduction, and that they should acquire the knowledge under the guidance of which alone the reasoning powers can be rightly exercised upon the phenomena of life and especially of disease. The peculiar excellence of this lecture consists in its plainness and simplicity, in the aptness of the illustrations afforded by its author's vast professional experience, and in the soundness of the advice which he has imparted to his young friends—equally applicable, however, to those in all periods of life who desire that their advance in knowledge should be coeval with its duration, as to the method in which they should study the complex problems that present themselves for investigation.

2. Dr. Guy's Lecture is chiefly remarkable for the force with which the introduction of scientific study as a branch of general education, and more especially as a preliminary to the prosecution of medical study, is advocated, both on the ground of its direct utility, and of its beneficial influence on the intellectual and moral faculties. We need not say that this view is most completely accordant with that which we have repeatedly urged ; and it gives us the greatest pleasure to find it so well enunciated in a college whose conservative tendencies might be supposed to make it look with a suspicious eye upon any show of a disposition to undervalue the pursuits of literature, or to substitute for the poetic contemplation of the past the practical claims of the present and the anticipated results of the future. That before science could be said to have an existence, and when all philosophy was to be found in the writings of the ancients, literature should be the chosen means of developing the higher powers of the intellect, is not surprising ; but that those who regulate the higher educational establishments of our country should still maintain so firm an adhesion to the wisdom of their ancestors, and should keep their eyes closed to the benefits which can be proved to result from an alteration of that system, speaks forcibly, we venture to think, as to their unfitness for the responsible trust of which they hold such firm possession.

3. The title of Mr. Paget's Lecture, whilst it truly indicates the principal subject of his address, does not convey an idea of the elevated manner in which that subject is treated; and we cannot deny ourselves the pleasure of quoting one or two passages which will show the high tone of feeling that he labours, and we hope not unsuccessfully, to excite in his pupils. After speaking of the peculiar demand for the energetic and persevering use of our best faculties which the study of medicine involves, he thus continues :

"Yield to the demand, and give them. For there is not in this world a nobler spectacle than that of a rational being devoting himself, with patient, earnest perseverance, to the cultivation of his powers, that they may be employed in the discharge of duty Knowing that a force within him is capable of unlimited expansion, and confessing in his inmost consciousness, that its development and its exercise are duties of strongest obligation, he pauses not to ask whether outward reward will crown his work or not ; much less, with scrupulous calculation, does he count the cost and gain. But, because he knows that the powers and opportunities he has received were given him for use, he resolves that not one of them shall run waste or wild : for him, to be indolent, were to be unthankful. And so, in toil, yet not in weariness, he pursues his way ; sowing seed, of which he reckons not whether he shall reap the fruit; content, because he is in the path of duty ; blest, if only he may see or think that he ministers to the welfare of his fellow-men. (p. 11.)

But the labour of acquisition, if rightly directed, is in itself a pleasure, as Mr. Paget eloquently urges. After pointing to the movements of animals as signs of the pleasure of energy, he continues :

"Now, there is an exact analogy here between the mental and the bodily faculties. In health, every exercise of the mind, provided it be voluntary and natural, is a true source of pleasure. And, therefore, we should count it as a privilege, that the pleasures of intellectual activity are offered to us in all their various forms in the study of our science ; for therein, we may always have the calm and abiding satisfaction which attends the gradual acquirement of knowledge; and, not seldom, that intenser pleasure which is perceived when difficulties, long striven against, are overcome ; and sometimes, if we carry our researches beyond the limit of that which is already known, we may enjoy the same excitement and expectation as others pursue in more perilous adventure ; and, then, we may attain the thrills of delight which accompany the first perception, and the slow unfolding, and, at last, the clear and perfect view, of some new truth or principle. And all these pleasures we may enjoy as long as we continue our study ; for the science is inexhaustible, and the pleasure becomes more intense in the same proportion as the faculties that are exercised are higher, and as the mind is more guided and illustrated by knowledge." (pp. 16-7.)

The responsibilities of the practitioner are equally well set forth in the pages immediately preceding that last quoted from. We greatly regret that we can find room for only a portion of the beautiful passage:

"We sometimes see the beam of life and death so nearly balanced, that it turns this way or that, according to the more or less of skill that can be cast into the scale of life. And surely, if we could gather into thought all the issues that are involved in the life or death of any man, the anxiety of ignorance at such a time should be intolerable. For at all such times, the issues and the responsibilities are manifold; it is not alone the fate of the sufferer (though in that, indeed, may be the most fearful consequence of all), but, as each of us must have felt in some instance very near to his own heart, those that stand around have all their various griefs and fears, their hopes, yet sad forebodings. And now, all is permitted to depend upon the skill of one. Conceive that one yourself: what would be your remorse, if, when in their confusion and distress they look to you, you feel helpless as themselves, utterly unworthy of the confidence with which they still lean on you; your hand paralysed by the fear of ignorance, your mind confused in that half-knowledge, whose glimmerings only show that more skill might save the dying man! Yet this must be the remorse of every one who will neglect the study of his profession, and yet dare to undertake its responsibilities." (pp. 13-4.)

"Do not imagine that your responsibilities will be limited to the events of life or death. As you visit the wards of this Hospital, mark some of the hardly less portentous questions which, before a few years are past, you may be permitted to determine. In one, you will find it a doubt whether the remainder of the patient's life is to be spent in misery, or in ease and comfort; in another, whether he, and those who depend upon his labours, are to live in hopeless destitution, or in comparative abundance. One who used to help his fellow-men, finds ground to fear that he may be a heavy burthen on their charity. Another counts the days of sickness, not more by pain and weariness, than by the sufferings and confusion of those who are left at home without a guide, and, it may be, starving. Oh! gentlemen, I can imagine no boldness greater than this would be, who would neglect the study of his profession, and yet venture on the charge of interests like these; and I can imagine no ambition more honorable, no envy so praiseworthy, as that which strives to emulate the acquirements of those who are daily occupied in giving safe guidance through the perilous passages of disease, and who, in all these various difficulties and dangers, can act with the energy and calmness that are the just property of knowledge." (pp. 14-5.)

